# Finding genomic ontology terms in text using evidence content

**DOI:** 10.1186/1471-2105-6-S1-S21

**Published:** 2005-05-24

**Authors:** Francisco M Couto, Mário J Silva, Pedro M Coutinho

**Affiliations:** 1 Departamento de Informática, Faculdade de Ciências da Universidade de Lisboa, Portugal; 2 Architecture et Fonction des Macromolécules Biologiques, CNRS, Marseille, France

## Abstract

**Background:**

The development of text mining systems that annotate biological entities with their properties using scientific literature is an important recent research topic. These systems need first to recognize the biological entities and properties in the text, and then decide which pairs represent valid annotations.

**Methods:**

This document introduces a novel unsupervised method for recognizing biological properties in unstructured text, involving the evidence content of their names.

**Results:**

This document shows the results obtained by the application of our method to BioCreative tasks 2.1 and 2.2, where it identified Gene Ontology annotations and their evidence in a set of articles.

**Conclusion:**

From the performance obtained in BioCreative, we concluded that an automatic annotation system can effectively use our method to identify biological properties in unstructured text.

## Background

The annotation of biological entities with their properties using text mining systems is an important issue amongst biological databases [1-3]. These annotations are crucial to improve the work of microarray, mass spectrometry and other communities. As an example, the GOA (Gene Ontology Annotation) project aims at identifying GO (Gene Ontology) annotations to supplement the UniProt knowledgebase [4]. They provide high-quality manual GO annotations but manual curation is a time-consuming task and currently covers only about 2.6% of UniProt. Thus, the GOA database coverage mainly consists of electronic annotations, which have a lower quality than manual annotations. One approach to improve their quality is the use of text mining systems, since, besides the identification of annotations, these systems can also locate their evidence in literature. The quality of the annotations extracted by a text mining system is directly related to its ability to recognize and locate in the texts the biological entities and their properties. Therefore, the recognition of biological entities and properties in text has become an important research topic.

This document presents FiGO (Finding Genomic Ontology), a novel unsupervised method to identify biological properties organized in a genomic ontology in unstructured text. FiGO uses the evidence content of each word present in the nomenclature of the ontology. This evidence content is inversely proportional to the number of times the word appears in the names of all properties. Our definition of evidence content derives from the definition of information content made by Resnik [5]. We assume that the evidence content of a word measures its importance to identify a property in text. For instance, consider the Gene Ontology (GO) term 'punt binding'. If only the word 'binding' is present in the text, the probability of the GO term being referred is very low, because 'binding' is used in many other names. On the other hand, if only the word 'punt' is present, then we have strong evidence that the GO term is mentioned in the text, because this word is not part of any other name. We evaluated FiGO in BioCreative tasks 2.1 and 2.2. This document describes its implementation details, and presents and discusses the results achieved.

## Methods

### FiGO

FiGO receives i) an ontology *Ont *and ii) a piece of text, *Txt*, as input. Each entry in *Ont *represents a biological property that can be assigned to biological entities. The output is the list of properties that FiGO detected in the given text. FiGO returns these properties ranked according to their likelihood of being mentioned in the text. For example, *Ont *can be the GO with each biological property representing a GO term, and *Txt *can be a sentence taken from a document.

#### The words

FiGO derives a map between the properties and their names:

*Names *(*prop*) = {*n*_0_,..., *n*_*k*_},

where *prop *∈ *Ont *and *n*_0_,..., *n*_*k *_are its name and synonyms in the ontology. If *prop *does not have synonyms, then *k *= 0 and *Names*(*prop*) = {*n*_0_}. The set of words that compose a name *n *is given by:

*Words*(*n*) = {*w*_0_,...,*w*_*l*_}.

In addition, we define the set of words contained in an property *prop *as:

*Words*(*prop*) = {*w *∈ *Words*(*n*) : *n *∈ *Names*(*prop*)}

Furthermore, the words of the ontology are

*Words*(*Ont*) = {*w *∈ *Words *(*prop*) : *e *∈ *Ont*}

#### Evidence content

The evidence content of each word decreases with its frequency. The frequency of a word *w *is the number of properties that contain the word:

*Freq*(*w*) = #{*prop *∈ *Ont *: *w *∈ *Words*(*prop*)}.

A word present in only one name has high evidence content. On the other hand, the word with the maximum frequency has no evidence content. The maximum frequency is defined using the following equation:

*MaxFreq *= *max*{*Freq*(*w*) : *w *∈ *Words*(*Ont*)}.

Thus, *WordEC*(*w*), the evidence content of a word *w*, is defined using the following equation:



Since each name is composed of a set of words, we can define the evidence content of a name *n *as the sum of the evidence content of its words:



The evidence content of an property *prop *is defined as the highest evidence content of all its names:

*EC*(*prop*) = *max*{*NameEC*(*n*) : *n *∈ *Names*(*prop*)}.

#### Local evidence content

The input text is transformed into a set of words:

*Txt *= {*w*_0_,...,*w*_*l*_}.

The local evidence content (LEC) is used to measure the likelihood that a given name *n *is mentioned in the text *Txt*. LEC is the sum of the evidence content of those words, which are present in the text as well as in the name:



The LEC is also used to measure the likelihood that a given property *prop *is mentioned in the text *Txt*:

*LEC*(*prop*, *Txt*) = *max*{*NameLEC*(*n*, *Txt*) : *n *∈ *Names*(*prop*)}.

The LEC divided by the EC is a confidence level for the property *prop *occurring in the *Txt*:



*Con f *(*prop*, *Txt*) ∈ [0, 1], since LEC is smaller than EC by definition.

If the confidence level is larger than a given threshold α ∈ [0, 1], then *prop *is considered to occur in *Txt*:

*Conf *(*prop*, *Txt*) ≥ α.

If α = 1, the complete name has to appear in the text to be selected. Thus, the α parameter is used to tune recall and precision of FiGO. An increase in α increases precision, a decrease in α increases recall. *Con f *(*prop*, *Txt*) is used to rank the returned properties, and represents the likelihood of each biological property occurring in text.

#### Example

Given a property *prop *with *Names*(*prop*) = {'punt binding','punt function'}, and *Freq*('*punt'*) = 1, *Freq*('binding') = 4, *Freq*('function') = 8, and *MaxFreq *= 16. Then, *WordEC*('*punt'*) = -*log*(1/16) = 4, *WordEC*('binding') = -*log*(4/16) = 2, *WordEC*('function') = -*log*(8/16) = 1, *WordEC*('punt binding') = 4 + 2 = 6, *WordEC*('punt function') = 4 + 1 = 5, and *EC*(*prop*) = *max*{6, 5} = 6. Considering the following pieces of text: *Txt*_1 _= 'The protein has a binding activity', *Txt*_2 _= 'The protein has a punt activity', and *Txt*_3 _= 'The protein has a punt binding activity', since *LEC*(*prop*, *Txt*_1_) = 2, *LEC*(*prop*, *Txt*_2_) = 4 and *LEC*(*prop*, *Txt*_3_) = 6. Then we have *Conf*(*prop*, *Txt*_1_) = 1/3, *Conf*(*prop*, *Txt*_2_) = 2/3 and *Con f *(*prop*, *Txt*_3_) = 1, which means that FiGO will decide that *prop *occurs in *Txt*_1 _when α ≤ 1/3, in *Txt*_2 _when α ≤ 2/3, and in *Txt*_3 _when α ≤ 1. By comparing the case of *Txt*_1 _and *Txt*_2_, we can realize how FiGO gives more importance to infrequent words to identify the properties in a given text.

### BioCreative application

This section describes the FiGO implementation used when preparing our submission to whose output we submitted to BioCreative tasks 2.1 and 2.2. Given an article and a GO annotation, task 2.1 consisted of identifying the text in the article that provided evidence for the annotation. Given an article and the number of GO annotations to find for each GO class, task 2.2 consisted of identifying the GO annotations and extracting a section of evidence text for each of them from the article.

#### GO pre-processing

In our implementation, we used the GO genomic ontology, considering its terms as the properties to identify. FiGO identified the set *Words*(*GO*), and removed from this set all the stop words, such as 'in' or 'on'. FiGO then computed the evidence content of each word, name, and finally of each term. FiGO also computed the annotation frequency of each GO term as the number of times the term and its descendants in the GO hierarchy were annotated in GOA. The most frequently annotated terms represent general GO terms, such as 'protein', and 'binding'. These terms were discarded in the extraction of annotations from text.

#### The text

FiGO parsed the SGML file given for each article and structured the text in sentences. Each sentence represented a piece of text from where FiGO identified GO terms.

In task 2.1, we selected from the ranked list of sentences returned by FiGO the ones where the given term occurred. In the case of having multiple sentences, we selected the one with the highest rank and also mentioning the protein. In the case of not having any sentence, we returned a sentence for the most similar term. To calculate the similarity between terms, we used FuSSiMeG [6]. In this task, we executed FiGO three times with α assigned to 0.3, 0.7 and 0.9, resulting in three different submissions.

In task 2.2, we selected from the ranked list of sentences returned by FiGO the ones mentioning the protein. Then, we discarded the generic terms by selecting the sentences containing the most infrequent annotated terms. In this task, we executed FiGO three times with the α assigned to 0.5, 0.7 and 0.9, resulting in three different submissions.

To identify the proteins in the text, we applied a naïve method based on exact matching. Given a sentence we consider that it mentions a protein if it contains all the words of its name or synonym. We collect the name and synonyms of each protein from UniProt database.

## Results

In the BioCreative task 2, each submitted prediction had a GO term and a protein evaluation. Both evaluations assigned a high, generally or low score to the prediction. High score means that the predicted evidence supports a correct GO term or protein. Generally score means that the predicted evidence supports a related GO term or protein. Low score means that the predicted evidence does not support a correct GO term or protein. A prediction was considered perfect when both the GO and protein evaluation assigned a high score to it.

**Figure 1:**
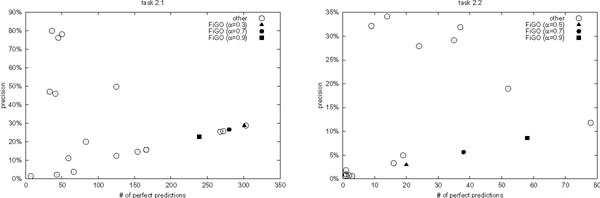
**Performance of all the submissions in tasks 2.1 and 2.2. **These charts compare the quality of the predictions returned by FiGO with all the other submissions to BioCreative tasks 2.1 and 2.2. For each submission, the charts show the precision versus the number of perfect predictions identified. The precision is the number of perfect predictions over the number of predictions submitted.

Figure 1 shows the performance of FiGO in tasks 2.1 and 2.2 by comparing its precision and number of perfect predictions with all the other submissions. In task 2.1, the best performance of FiGO was obtained using α = 0.3, which achieved a large number of perfect predictions and a precision of almost 30%. On the other hand, in task 2.2 the best performance of FiGO was obtained using α = 0.9, which achieved a significant number of perfect predictions and precision of almost 10%.

**Figure 2:**
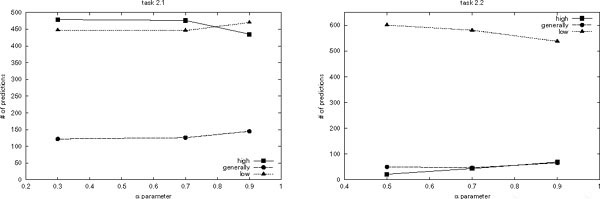
**GO evaluation of FiGO predictions in tasks 2.1 and 2.2. **A prediction was considered perfect when it provided high evidence of both GO term and protein. Thus, a prediction could provide high evidence of the GO term without being perfect. These charts disregard the protein evaluation and show the number of predictions submitted by FiGO to BioCreative tasks 2.1 and 2.2, which provided a high, generally and low evidence of the GO term for each value of α used.

Figure 2 shows the GO evaluation of FiGO predictions for the values of α used in tasks 2.1 and 2.2. The charts show the number of predictions that provided high, generally, and low evidence of the GO term regardless of the protein evaluation. The manipulation of the α parameter had a different impact on each task. In task 2.1, we obtained better results using a smaller α value. On the other hand, in task 2.2 the increase of α implied a better performance of our approach.

**Figure 3:**
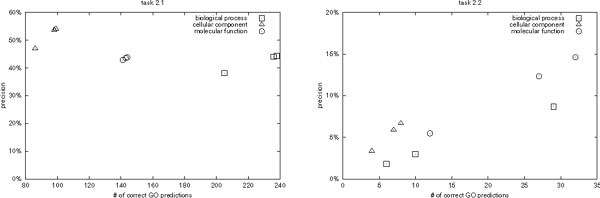
**Performance of FiGO in each class of the GO hierarchy in tasks 2.1 and 2.2. **For each class of the GO hierarchy, these charts compare the performance of the three submissions of FiGO to BioCreative tasks 2.1 and 2.2. For each class, the charts show the precision versus the number of correct GO predictions identified by each submission. The precision is the number of correct GO predictions over the number of predictions submitted.

Figure 3 compares the performance of FiGO in each class of GO. The charts show the precision and number of correct GO predictions obtained by our submissions to tasks 2.1 and 2.2. In the figure, a prediction was considered a correct GO prediction when the GO evaluation assigned a high score to it. In task 2.1, the best performance of FiGO was in the biological process class. On the other hand, in task 2.2 the best performance of FiGO was in the molecular function class.

## Discussion

FiGO achieved a good performance when compared to the other submissions. In both tasks, FiGO almost defined the highest number of correct predictions, but its precision was far from the best results. However, the submissions with higher precision were composed by fewer predictions that requested. We chose to always submit the requested number of predictions, even when they had a low confidence score. Since the core of FiGO was the identification of GO terms, a significant part of our predictions was not considered perfect just because of the protein evaluation. For example, in task 2.1 with α = 0.3, the GO evaluation assigned a high score to 479 predictions (see Figure 2). However, only 301 of them were considered perfect (see Figure 1). This means that 178 out of 479 predictions (37.2%) were not considered perfect because they did not provide high evidence of the protein. In addition to this major problem, we also identified the following problems in FiGO:

• in task 2.1, it predicted about 20 obsolete GO terms;

• it did not filter the GO terms that could not be annotated with Human proteins (e.g. germination);

• it selected sentences from irrelevant sections (e.g. 'Material and Methods');

• sometimes just one sentence is not enough to support an annotation. For instance, when the protein and the term are in the same paragraph, but not in the same sentence;

• it did not take in account the number of times a term occurs in the text;

• it did not take in account the word order in the name;

• in task 2.2, it predicted GO terms out of context.

The first two problems could be easily solved before BioCreative, but we were not able to identify them at that time. On the other hand, the last five problems represent important topics of research that deserve further study by the research community. The performance in task 2.2 was lower than in task 2.1 mainly because of the last problem on the list. In our opinion, to discard terms out of context we have to use some domain knowledge about the proteins and the articles. For instance, in *KDD2002 Cup challenge: bio-text task*, statistical text classification systems reasoning without considering domain knowledge achieved also poor results [7]. An effective approach is to obtain the required domain knowledge from publicly available resources [8].

In task 2.1, the GO terms with higher precision occurred in the literature exactly as described in GO, such as 'cell proliferation'. This particular GO term had the highest precision with 11 high and 1 low scores assigned. The GO terms with lower precision were the ones whose name was composed by words with low evidence content, such as 'regulation of transcription'. This particular GO term had the lowest precision with 1 high and 8 low scores assigned.

In task 2.2, the GO terms with higher precision were generic terms, such as 'binding'. Those whose name had high evidence content, such as 'galactose 3-O-sulfotransferase activity'. This last GO term had the second highest precision with 4 high and 2 low scores assigned. The GO term 'binding' had the highest precision with 20 high and 3 low scores assigned. The GO terms with lower precision were the ones whose name was composed by words with low evidence content or multiple meanings, such as 'receptor activity'. This particular GO term had the lowest precision with 1 high and 8 low scores assigned, because 'activity' has low evidence content and 'receptor' can be used to mention other protein. For example, in UniProt there are more than 20000 proteins whose name contains the word 'receptor'.

From Figure 3, we concluded that in task 2.1 it was easier to find evidence for GO terms from the biologic process. This can be explained because these terms use very specific names. On the other hand, we conclude that in task 2.2 it was easier to predict terms from the molecular function class. This can be explained because normally we can find more occurrences of these terms in the articles.

The reason for having better results using a smaller α value in task 2.1 is that there were a large number of terms not explicitly mentioned in the text. Some sentences were correctly selected when only less than 70% of the term's name appeared in text. On the other hand, for smaller values of α, FiGO identified more terms out of context. Thus, in task 2.2, the selection of terms with a larger α turned up to be an effective approach to predict which relevant terms were mentioned.

## Conclusion

This document presents FiGO, a novel unsupervised method for recognizing biological properties in unstructured text, involving the evidence content of their names. FiGO does not need training data, since it computes the evidence content based on the nomenclature of a genomic ontology that structures the properties. Therefore, the use of FiGO represents little human intervention.

FiGO was designed for recognizing properties and not for extracting annotations, but besides that FiGO obtained a good performance in BioCreative when compared to other submissions. From the results, we identified a set of problems that should be addressed in the next implementation. The main problem of our predictions was the protein identification. If instead of implementing a naïve method we used a more effective method, FiGO would have achieved a higher performance.

The performance of FiGO demonstrates that it provides an effective approach to recognize properties in scientific literature, improving the performance of automatic annotation systems.

## Authors' contributions

FMC conceived this study, designed the methods introduced, and implemented them. MJS and PC coordinated this study and performed an analysis of the results obtained. All authors collaborated since the beginning of the project. All authors read and approved the final manuscript.
